# External apical root resorption after orthodontic treatment: analysis in different chronological periods

**DOI:** 10.1590/2177-6709.27.5.e2220100.oar

**Published:** 2022-11-07

**Authors:** Bruno Moreira das NEVES, Luciana Quintanilha Pires FERNANDES, Jonas CAPELLI

**Affiliations:** 1 Universidade do Estado do Rio de Janeiro, Faculdade de Odontologia, Departamento de Odontologia Preventiva e Comunitária (Rio de Janeiro/RJ, Brazil). bruno_moreira_moreira@hotmail.com

**Keywords:** Root resorption, External apical root resorption, Orthodontics

## Abstract

**Introduction:**

External apical root resorption (EARR) is characterized by the definitive loss of tooth root structure, with a higher incidence in lateral and central maxillary incisors.

**Objective:**

To identify, in different chronological periods, the incidence of EARR in the maxillary incisors (MI) of patients orthodontically treated with or without premolars extraction.

**Methods:**

Periapical radiographs before and after orthodontic treatment of 1,304 MIs from 326 patients (205 women and 121 men) were evaluated for EARR, divided into five groups, according to the chronological period in which treatments were started: G90) from 1990 to 1994, G95) from 1995 to 1999, G00) from 2000 to 2004, G05) from 2005 to 2009, G10) from 2010 to 2015. The evaluation was performed in each group, in patients who underwent maxillary first premolars extraction and those who did not. For statistical analysis, Fisher’s exact test was used, with a significance level of *p* < 0.05. The EARR was measured using the adapted Levander and Malmgren classification.

**Results:**

Incidence of EARR was higher in MIs of patients treated with maxillary premolar extraction (*p* < 0.05) in two chronological periods (G00 and G10), also being influenced by orthodontic treatments with longer duration, and due to possible individual genetic factors.

**Conclusion:**

Even with the limitations of a retrospective study, the lack of a defined EARR pattern in the MIs at different chronological periods was larger in the experimental group, due to the sum of factors such as premolars extraction, prolonged orthodontic treatment, possible genetic characteristics, and root shape, without the influence of the sex and age.

## RESUMO

**Introdução:**

A reabsorção radicular apical externa (RRAE) é caracterizada pela perda definitiva da estrutura dentária na região radicular, com maior incidência em incisivos centrais e laterais superiores.

**Objetivo:**

Identificar, em diferentes períodos cronológicos, a incidência de RRAE em incisivos superiores (IS) de pacientes tratados ortodonticamente com e sem extração de pré-molares.

**Métodos:**

Por meio de radiografias periapicais antes e após o tratamento ortodôntico, 1.304 IS de 326 pacientes (205 mulheres e 121 homens) foram avaliados quanto à RRAE, divididos em cinco grupos, de acordo com o período cronológico em que os tratamentos foram iniciados: G90) de 1990 a 1994; G95) de 1995 a 1999; G00) de 2000 a 2004; G05) de 2005 a 2009; e G10) de 2010 a 2015. Em cada grupo, foi realizada avaliação nos pacientes submetidos à extração dos primeiros pré-molares superiores e nos que não foram. Para análise estatística, foi utilizado o teste exato de Fisher, com *p*< 0,05; a RRAE foi mensurada por meio da classificação de Levander e Malmgren modificada.

**Resultados:**

A incidência da RRAE foi maior em IS de pacientes tratados com extrações de pré-molares (*p*< 0,05) em dois períodos cronológicos (G00 e G10), além de ter sido influenciada por tratamentos ortodônticos de maior duração e por possíveis fatores genéticos individuais.

**Conclusão:**

Mesmo com as limitações de um estudo retrospectivo, a ausência de um padrão definido de RRAE nos IS nos diferentes períodos cronológicos foi maior no grupo experimental, devido à soma de fatores, como extração de pré-molares, tratamento ortodôntico prolongado, possíveis características genéticas e morfologia radicular, sem influência do sexo e da idade.

## INTRODUCTION

Orthodontics has changed conduct throughout history. After Angle’s death, one of his followers, Charles Tweed, evaluated previously treated cases without extractions and opted to retreat cases with relapses. Analyzing the cases treated without extractions, as recommended by Angle, he observed that 80% of the patients did not have adequate stability, facial aesthetics, periodontal health, and function. From that moment on, Tweed started to advocate extractions as an alternative to obtain facial harmony and greater post-treatment stability.^1^

Due to these previously approached issues regarding stability, aesthetics and function after orthodontic treatments with extractions, tooth extractions for orthodontic reasons started to be more performed at the end of the 1940s.^2^

Orthodontics finds itself in a conservative era, in which the tendency is to conduct treatments without extractions. Despite this non-extraction tendency, when correctly indicated, tooth extractions for orthodontic reasons are still considered the most appropriate therapeutic solution for some cases.^3^

External apical root resorption (EARR) has been associated with orthodontic treatment, and is considered a collateral effect that culminates in the permanent and irreversible loss of tooth structure (dentin and/or cementum)^4^. Orthodontic forces with different magnitudes have been associated to the incidence of EARR, as well as the severity it affects the teeth.^5^ ^,^ ^6^ EARR can occur in any tooth during orthodontic treatment, being the maxillary lateral and central incisors the most frequently affected ones.^7^

Levander and Malmgren^8^ evaluated initial and final periapical radiographs of patients undergoing orthodontic treatment with a fixed appliance and classified the severity of EARR in five different levels, ranging from the absence of resorption to extreme resorption. 

Regarding patients treated with conventional fixed appliances, more than a third of them usually have root resorption up to 3 mm.^9^ Severe EARR is characterized by a loss of 5 mm of root length, and affects about 2% to 5% of orthodontic patients, imposing a risk to the function and maintenance of the resorbed tooth.^10^

In orthodontic treatment, when the mechanical forces are interrupted, the EARR process also ceases; however, resorption can return and progress if tooth movement restarts, due to the application of forces.^11^

A systematic review^12^ showed that the application of forces at increased levels has a positive correlation with the increase in the amount of root resorption; as well as more prolonged treatments are related to greater resorption. In addition to these factors, a pause in tooth movement can be beneficial in these cases, because it allows the healing of the reabsorbed cement.

EARR is a consequence of an inflammatory process and presents some factors that may be related to its severity, such as: root shape, dental trauma, endodontic treatment, genetic predisposition,^13^ age,^14^ use of mechanical forces to perform orthodontic movements, and the duration of orthodontic treatment.^7^

Thus, the present study aimed to evaluate the incidence of EARR on maxillary incisors (MI), in orthodontic treatments performed with or without extractions, in five different chronological periods, from 1990 to 2015, at the State University of Rio de Janeiro (UERJ, Brazil).

## MATERIAL AND METHODS

In this unicenter retrospective study, in which a convenience sample was used, the documentation of 434 patients was evaluated, among which, 326 (205 women and 121 men, with an average age of 15.55 years at the beginning of treatment) met the inclusion criteria: present anamnesis form and history of all procedures performed during orthodontic treatment; presence of the four MIs; for patients in the experimental groups (EG), absence of teeth #14 and #24, due to orthodontic reasons; for patients in the control groups (CG), the presence of teeth #14 and #24; initial and final periapical radiographs of the four MIs, with the final radiograph acquired no more than six months after the end of active orthodontic treatment; the patients should be in the retention phase, after orthodontic treatment performed at UERJ. The exclusion criteria were: dental trauma history in the MI; the presence of restoration on the incisal edge; endodontic treatment; incomplete root formation; incomplete or previous corrective orthodontic treatment; systemic disorders or syndromes; absence of any MI.

**Figure 1:**
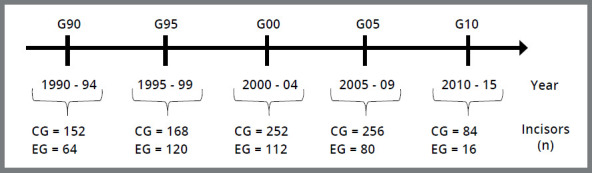
Timeline representing the groups division: Chronological periods of orthodontic treatment (G90, G95, G00, G05, G10) and their respective control (CG) and experimental (EG) groups.

After analyzing the inclusion and exclusion criteria, 1,304 MIs were used, corresponding to the number of MI of the patients selected for the evaluation of the EARR. The four MIs were selected for the study due to the greater susceptibility to root resorption, as reported in the literature.^7^ ^,^ ^15^ These 1,304 MIs were allocated into five groups, according to the chronological period in which orthodontic treatment was started: G90) from 1990 to 1994, G95) from 1995 to 1999, G00) from 2000 to 2004, G05) from 2005 to 2009, G10) from 2010 to 2015. A CG was established within each of these five groups, in which extractions of teeth #14 and #24 were not performed for orthodontic reasons; and an EG, in which extractions of teeth #14 and #24 were performed for orthodontic reasons (Fig 1). 

### MEASUREMENT OF EXTERNAL APICAL ROOT RESORPTION

**Figure 2:**
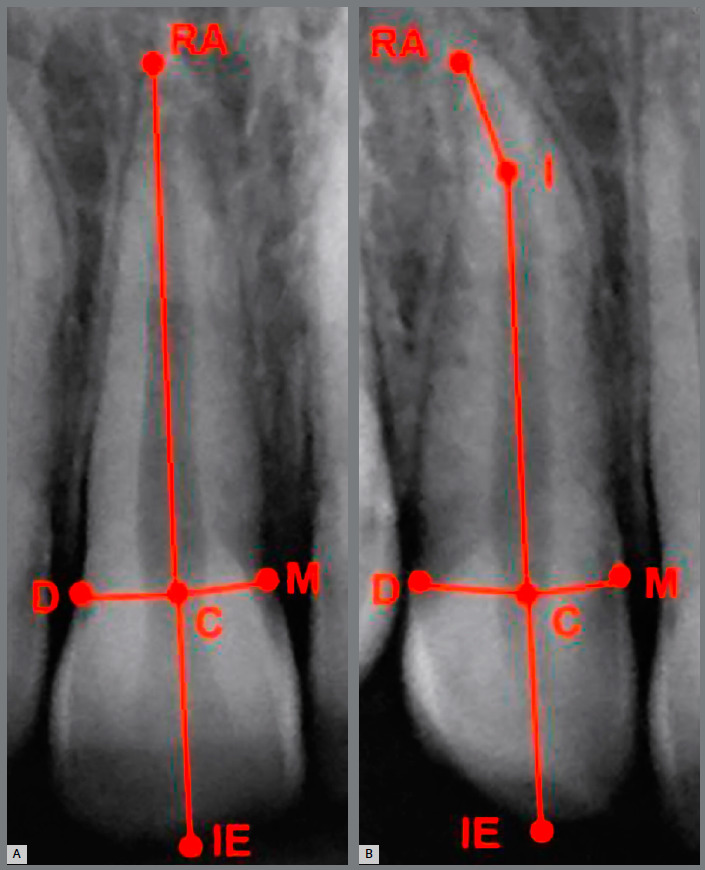
Reference points for measuring root length. Points marked in (A) rhomboid, triangular root, with pipette shape; and (B) dilacerated root: **RA** = root apex; **M** = mesial point of the CEJ; **D** = distal point of the CEJ; **C** = central point of the line that joins M and D; **I** = point of intersection of the long axis of the tooth, starting from C, and the long axis of the dilacerated root portion, starting from RA.

**Figure 3:**
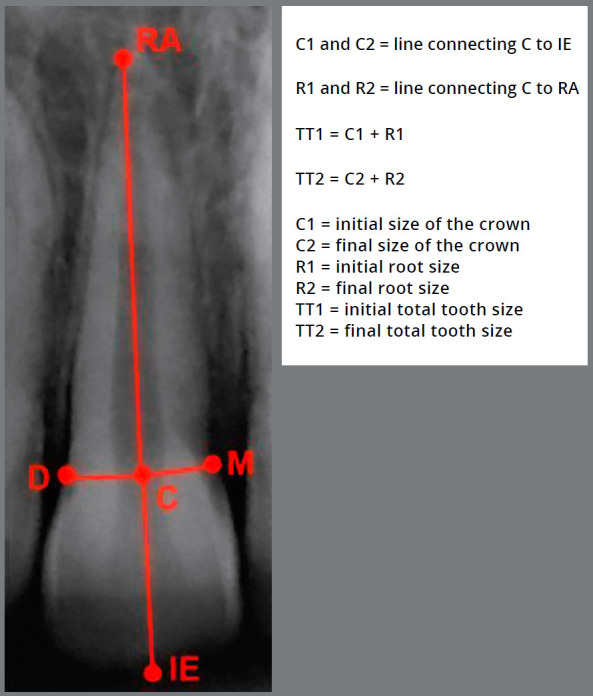
Points and lines used to measure the EARR: **RA** = root apex; **M** = mesial point of the CEJ; **D** = distal point of the CEJ; **C** = central point of the line that joins M and D; **IE** = central point of the incisal edge.

Images of the periapical radiographs for the evaluation of EARR in the MIs were obtained using the method described by Fernandes et al^16^, in which the initial and final periapical radiographs were digitized with 300 dpi resolution and 256 gray levels (Scanjet 4890; Hewlett-Packard, Palo Alto, CA, USA) and saved in JPEG (Joint Photographic Experts Group) format. For the measurements to be performed, the radiographs were imported to the Image J software (National Institutes of Health, Bethesda, MD, USA). To calibrate the image size, the size of the radiographic film (40 mm) was used as a reference measure. At the time of measurement, the examiners were blinded regarding the time of the radiograph (initial or final). Finally, after calibrating and obtaining the images, measurements and evaluations of EARR were performed in the MIs, by the method described by Linge and Linge,^7^ in which initial periapical radiographs were used to collect data regarding root length, by means of the following measurements: 

1) Crown size - measured from the central point of the incisal edge to the central point of the cementoenamel junction (CEJ). This measurement was performed in two stages: C1 (before orthodontic treatment, measured on the initial radiograph) and C2 (after orthodontic treatment, measured on the final radiograph). 

2) Root size - measured from the central point of the CEJ line to the root apex, following the long axis of the tooth. This measurement was performed in two stages: R1 (before orthodontic treatment, measured on the initial radiograph) and R2 (after orthodontic treatment, measured on the final radiograph) (Fig 2). In cases of dilacerated root, the following measures were summed: from the central point of the CEJ line to the point of intersection between the long axis of the tooth and the dilacerated root portion, and from this point to the root apex, as shown in Figure 2. 

3) Total tooth size: this measure was obtained with the sum of C1 + R1 and C2 + R2, resulting in the measures TT1 (total tooth size before orthodontic treatment) and TT2 (total tooth size after orthodontic treatment), as can be seen in Figure 3.

For the calculation and subsequent classification of EARR, the formula described by Linge and Linge^7^ was used: R1-R2 [C1/C2], in which the amplification factor is defined by C1/C2, assuming that the crown size did not change during the treatment.

### CLASSIFICATION OF INCISORS ACCORDING TO EARR

Each of the 1,304 MIs was measured for EARR using the Image J software (National Institutes of Health, Maryland, USA). After being measured, the classification proposed by Levander and Malmgren^8^ was used, with some modifications. The initial classification of EARR would score the degree of resorption in five different levels: level 0 = absence of resorption, with no change in the root apex; level 1 = minimal resorption, with changes in the root apical contour; level 2 = moderate root resorption up to 2 mm; level 3 = severe root resorption greater than 2 mm and less than 1/3 of the root length; level 4 = extreme resorption greater than 1/3 of the root length. The classification used to analyze the data in the present study was determined as follows: the MIs scored as level 0 or 1 according to the classification proposed by Levander and Malmgren^8^ were considered as incisors with no EARR; and the incisors scored as level 2, 3, or 4 were classified as incisors affected by EARR.

### STATISTICAL ANALYSIS

The software Statistical Package for Social Sciences v. 23.0 (SPSS Inc., Chicago, IL, USA) was used for data analysis.

As this was non-parametric data, it was not necessary to verify the normality of the sample,^17^ and the data were then characterized as non-normal distribution with more than two groups, with independent samples. Considering the needs of the described sample, Fisher’s exact test was selected to assess whether there was a difference in the EARR in the MIs in the different chronological periods in patients who had undergone maxillary first premolars extraction, considering the power at 95% with a significance level of 5%.

## RESULTS

In the descriptive analysis of the data, 1,304 incisors were evaluated, 652 central incisors and 652 lateral incisors. At the end of the data evaluation, 700 teeth (53.68%) were affected by EARR, while the other 604 teeth (46.32%) were not affected. Drawing a comparison between central and lateral incisors, the lateral incisors were more affected by EARR (62%) than the central incisors (49.9%). Regarding the shape of the roots, 1,304 roots were evaluated, being possible to observe the distribution of root shape by chronological periods and groups in Table 1.

**Table 1:** Root shapes distribution, by chronological periods.

G90	CG (n = 152)					EG (n = 54)				
Root shape	#12	#11	#21	#22	%	#12	#11	#21	#22	%
Dilacerated	5	0	0	6	7.23	6	1	1	6	21.87
Rhomboid	22	23	23	23	59.8	7	12	12	8	60.93
Triangular	9	15	15	9	31.57	3	2	2	2	14.06
Pipette	2	0	0	0	1.31	0	1	1	0	3.12
G95	CG (n = 168)					EG (n = 120)				
Root shape	#12	#11	#21	#22	%	#12	#11	#21	#22	%
Dilacerated	9	3	4	9	14.88	8	0	0	11	15.8
Rhomboid	24	24	23	18	52.97	17	18	17	11	52.5
Triangular	7	13	12	11	25.59	5	10	10	3	23.3
Pipette	2	2	3	4	6.54	0	2	3	5	8.3
G00	CG (n = 252)					EG (n = 112)				
Root shape	#12	#11	#21	#22	%	#12	#11	#21	#22	%
Dilacerated	10	2	2	11	9.92	6	0	0	7	11.6
Rhomboid	37	39	37	37	59.52	11	15	15	10	45.53
Triangular	9	17	17	11	21.42	8	11	11	8	33.92
Pipette	7	5	7	4	9.12	3	2	2	3	8.92
G05	CG (n = 256)					EG (n = 80)				
Root shape	#12	#11	#21	#22	%	#12	#11	#21	#22	%
Dilacerated	15	0	1	17	12.89	7	0	0	6	16.25
Rhomboid	36	37	37	32	55.46	10	14	14	13	63.75
Triangular	9	23	21	10	24.6	3	6	6	1	20
Pipette	4	4	5	5	5.46	0	0	0	0	0
G10	CG (n = 84)					EG (n = 16)				
Root shape	#12	#11	#21	#22	%	#12	#11	#21	#22	%
Dilacerated	5	1	1	7	16.66	1	0	0	2	18.75
Rhomboid	11	13	14	11	58.33	3	3	3	2	68.75
Triangular	5	5	5	3	21.42	0	1	1	0	12.5
Pipette	0	2	1	0	3.57	0	0	0	0	0

#12 = tooth #12, #11 = tooth #11, #21 = tooth #21, #22 = tooth #22.

Taking into account that rhomboid roots were less affected by EARR than pipette-shaped and dilacerated roots, which were more affected by EARR,^18^ ^,^ ^19^ when analyzing the distribution of root shape in the different chronological periods, in the G00, the percentage of rhomboid roots was considerably higher in the CG than in the EG; making the CG teeth less susceptible to EARR (Table 1).

To analyze the data obtained in the statistical analysis related to the incidence, the EARR was evaluated on the four incisors of each patient: maxillary right lateral incisor (#12), maxillary right central incisor (#11), maxillary left central incisor (#21) and maxillary left lateral incisor (#22). There was no statistically significant difference (*p* > 0.05) in the EARR between CG and EG in three of the five chronological periods: G90, G95, and G05. In the G00, there was a statistically significant difference (*p* < 0.05), with more EARR in teeth #12, #11, #21, and #22 in EG, when compared to the CG. Finally, in G10 there was a statistically significant difference (*p* < 0.05), with a higher EARR only in tooth #21 in the EG, when compared to the CG (Table 2). The EARR affected different teeth, teeth of different patients and even different teeth of the same patient, that is, the EARR occurred without a defined pattern, since the sample presented variations regarding sex, ethnicity, age and root shape, even in the chronological periods different from the beginning of orthodontic treatment.

**Table 2:** Comparison of the EARR on MIs in the different chronological periods, in patients with (EG) and without (CG) extraction of first premolars.

Group	Tooth	EG EARR	EG Absence of EARR	CG EARR	CG Absence of EARR	p
G90	#12	12	4	23	5	0.365
	#11	7	9	12	26	0.534
	#21	8	8	17	21	0.772
	#22	11	5	20	18	0.370
Total		38	26	72	71	
G95	#12	17	13	23	19	1.000
	#11	16	14	19	23	0.633
	#21	15	15	19	23	0.812
	#22	20	10	22	20	0.332
Total		68	52	83	85	
G00	#12	23	5	32	31	0.005*
	#11	22	6	31	32	0.011*
	#21	23	5	28	35	0.001*
	#22	26	2	34	29	0.000*
Total		94	18	125	127	
G05	#12	12	58	37	27	1.000
	#11	9	11	29	35	1.000
	#21	11	9	25	39	0.301
	#22	14	6	40	24	0.603
Total		46	84	131	125	
G10	#12	1	3	8	13	1.000
	#11	1	3	8	13	1.000
	#21	4	0	8	13	0.039*
	#22	2	2	11	10	1.000
Total		8	8	35	49	

*Fisher’s exact test, significant at *p* < 0.05. #12 = tooth #12, #11 = tooth #11, #21 = tooth #21, #22 = tooth #22.

Two examiners were calibrated to assess the measurements: E1 (examiner 1) and E2 (examiner 2), for evaluating the intra-examiner and inter-examiner relationship. The Kappa test showed a substantial degree of agreement for intra-examiner E1 (89.1% and 0.774), and a degree of agreement near to perfection for intra-examiner evaluation of E2 (93.3% and 0.863), in addition to substantial agreement values inter-examiner (86.4% and 0.725), according to the classification proposed by Landis and Koch.^20^

**Figure 4:**
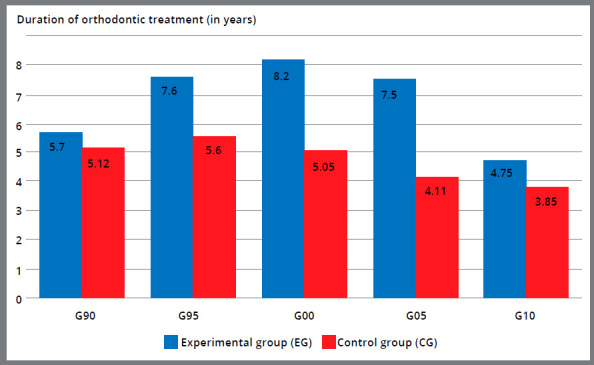
Comparison of the duration of orthodontic treatment, by chronological periods, in years.

Regarding the duration of treatment, the different chronological periods showed variations that should be considered in the evaluation of results: in these periods, treatments with extractions had a longer duration in EG, whereas in G00 the difference in treatment time was 3.15 years and in G05 was 0.9 years (Fig 4).

The treatments had a longer duration mainly in patients treated with extractions of premolars; however, the treatments performed without extractions in the different chronological periods also had a duration considerably longer than desired. The discussion of this topic shows the complexity of the different treatments performed in patients treated without extractions, since the Clinic of Specialization in Orthodontics at UERJ is a reference in orthodontic treatments of the highest degree of complexity in the state of Rio de Janeiro (Brazil). The types of treatments performed included, among others: Class II and III orthodontic-surgical treatment, anterior open bite, unilateral posterior crossbite, dental absences that required multidisciplinary treatment, and functional orthopedic treatments that required two phases.

It can be seen that from 1990 to 2000, the treatments with extractions showed a duration increase, and from 2005 onwards, there was a reduction in this time, reducing even more from the year 2010 (Fig 4). This variation can be credited to the duration of treatments in parts, to the differences in the conduction of the treatments, as well as the collaboration of each patient. It is important to highlight that the G10 group presented a smaller number of treated patients, due to the proximity to the time when this study was realized..

Taking into account the sex variable, the sample was heterogeneous, with different distributions according to chronological periods, as showed in Table 3.

**Table 3:** Sample distribution according to sex.

G90	Male (n)	%	Female (n)	%
CG	15	39.47	23	60.53
EG	6	37.5	10	62.5
G95	Male (n)	%	Female (n)	%
CG	18	42.85	24	57.15
EG	10	33.34	20	66.66
G00	Male (n)	%	Female (n)	%
CG	27	42.86	36	57.14
EG	11	39.28	17	60.72
G05	Male (n)	%	Female (n)	%
CG	21	32.81	43	67.19
EG	9	45	11	55
G10	Male (n)	%	Female (n)	%
CG	7	35	13	65
EG	2	50	2	50

CG = control group. EG = experimental group.

## DISCUSSION

During the development of this study, some factors that could influence EARR were evaluated, to try to explain the statistically significant higher incidence of EARR in patients in G00 and G10: in G00, teeth #12, #11, #21, and #22 were more affected by EARR in patients treated with maxillary first premolars extraction, as well as tooth #21 from patients in the G10. This could be because EARR has a multifactorial etiology that comes from a complex interaction between the effect caused by mechanical forces applied during active orthodontic treatment and the patient’s biology.^21^

The individual or biological characteristics of the patients mentioned in this study are more specifically related to the genetic component of each patient, as well as their genomic information, which will determine or will codify proteins and signaling mechanisms related to root resorption or repair of cementum and dentin during orthodontic treatment.^22^ ^,^ ^23^

The genetic component is related to the susceptibility to the development of EARR in patients submitted to external factors, such as mechanical forces applied to the teeth during orthodontic treatment. A limitation of this study, as it was a retrospective study, is the impossibility of obtaining material for the genetic evaluation of these patients. Among some studies that correlate EARR with the genetic component, some have observed its relationship with interleukin (IL): Lages et al^24^ showed that a variation of the IL1B gene (+3954) resulted in a greater risk of developing EARR; Gülden et al^25^ reported a relationship between IL1A (-889) and EARR. In addition to IL, other genes have also been correlated to EARR, such as P2RX7 (rs1718119).^26^ Even with the cited evidence of correlation, no defined genetic target has been widely selected to assist in predicting which patients are most susceptible to develop EARR during orthodontic treatment.^27^

The absence of a defined pattern of EARR in groups of teeth in the evaluated chronological periods leads us to believe that the EARR that affected the G00 incisors in a statistically significant way and a group of incisors in the G10, in patients who underwent maxillary first premolars extraction for orthodontic reasons, was mainly due to the individual characteristics of susceptibility to EARR of each patient, as genetic characteristics. This is based on the fact that other studies considered individual susceptibility as the main factor for EARR in patients submitted or not to orthodontic treatment^28^. Also corroborating the present results, another study^29^ reported that each patient has a different response to the applied mechanical forces, and may present different degrees of resorption in different teeth.

Considering the average age of patients at the beginning of treatment in different chronological periods, that gradually increased over the years (G90 = 12.98 years; G95 = 14.05 years; G00 = 14.13 years; G05 = 17.87 years and G10 = 18.70 years), studies^30^ ^,^ ^31^ have shown that there is no relationship between the patients’ age at the beginning of orthodontic treatment and the degree of EARR at the end of treatment. This goes against the results of the present study, which demonstrated statistically significant differences between the CG and EG in groups G00 and G10, which had, respectively, the third lowest and the highest average age, with a difference of 4.57 years.

Regarding the correlation between patient sex and EARR, the literature shows that there is no consensus and some authors have not observed differences in EARR between men and women.^15^ ^,^ ^32^ ^-^ ^34^ This goes against the present results, since in all chronological periods the number of women in the EG was always greater than or equal to the number of women in the CG, and not all groups showed differences between CG and EG. A meta-analysis^35^ reported that the duration of active orthodontic treatment and displacement of root apexes are highly related to an increase in the severity of EARR. In the present study, a factor that is believed to have influenced EARR is that some incisors were more poorly positioned before orthodontic treatment, and it was necessary to perform greater movements, subjecting these particular teeth to continuous orthodontic mechanical forces to correct their inadequate position. Thus, there may have been more displacement of the apexes in the groups where there was a statistically significant difference, resulting in a longer treatment time. This can be justified in the G00, which was the group in which the patients treated with extractions of the maxillary first premolars presented the highest average duration of treatment among all the chronological periods evaluated. It is worth noting that in G10, which also showed differences in terms of EARR, patients treated without extraction of premolars had the lowest average duration of orthodontic treatment among all the evaluated chronological periods. This can be based on the literature^35^ that supports the fact that, as treatments performed in the CG demanded a shorter duration, these teeth would be less susceptible to EARR.

Some authors^36^ ^,^ ^37^ demonstrated that there is a relationship between EARR and dental extractions in patients who have undergone orthodontic treatment. In the study by Fernandes et al,^16^ the authors concluded that the risk of developing EARR greater than 2 mm in MIs is 70% higher in patients treated with premolars extraction. In another study^38^ in which EARR was also evaluated in patients with and without extractions, patients treated with extractions of first premolars showed greater resorption in the MIs than those treated without extractions. In the present study, there was a significant difference in the occurrence of EARR in two chronological periods of orthodontic treatment beginning (G00 and G10), with greater EARR in some groups of teeth in EG patients, when compared to CG patients, corroborating the results from previously cited studies, which show that treatments with extractions influence EARR, when compared to treatments without extractions.^16^ ^,^ ^36^ ^-^ ^38^ Regarding the orthodontic mechanics used to perform the treatment of patients allocated to the EG, this factor could not be correlated to the EARR, considering that the EARR occurred without a defined pattern. Except for two cases, the retraction of the maxillary incisors and canines was performed in two phases, being the first phase for canines distalization with an elastomeric chain; and in the second phase, after the canines were already in the correct position, a 0.019 x 0.025-in retraction steel archwire was used to retract the four incisors. In the two cases in which the retraction was not performed in two phases, it was performed *en masse* (canines and incisors retracted at once) with a 0.019 x 0.025-in retraction steel archwire.

Another factor that may be related to EARR is root shape, taking into account that some studies^18^ ^,18,^ ^39^ showed that roots with normal shape are less affected than roots with shapes considered to be non-standard, such as pointed and dilacerated roots. This goes against some of the present results, in which G00 presented a percentage of rhomboid roots considerably higher in the CG than in the EG, making the CG teeth less susceptible to EARR - possibly due to their root shape. However, a systematic review^4^ relating the root shape to the EARR concluded that this variable does not seem to be related to the degree of resorption after the end of active orthodontic treatment. This strongly indicates the relationship between EARR that occurred in these groups of teeth, already mentioned in this study, and the individual susceptibility characteristics of each patient; and that there may have been the influence of the root shape in some patients, but it is not possible to say that this factor was determinant in the severity of EARR.

## CONCLUSION

Considering the lack of a defined pattern of EARR in the MIs evaluated during the studied chronological periods, the limitations of a retrospective study, and because the susceptibility characteristics of each patient could not be assessed - as possible genetic aspects-, the evaluation of different chronological periods was performed due to the technological evolutions and changes in concepts and techniques that frequently occur in Orthodontics, which could impact the incidence of EARR in the MIs of some patients. Thus, this article demonstrated that, in the chronological periods in which the incidence of EARR presented statistically significant differences between groups (G00 and G10), the patients treated with the extraction of premolars and with orthodontic treatments of longer duration were in the EG, and still had a lower amount of roots with rhomboid shape, when compared to the CG patients, without the influence of age or sex. 
